# Impact of the condolence letter on the experience of bereaved families after a death in intensive care: study protocol for a randomized controlled trial

**DOI:** 10.1186/s13063-016-1212-9

**Published:** 2016-02-20

**Authors:** Nancy Kentish-Barnes, Sylvie Chevret, Elie Azoulay

**Affiliations:** Saint-Louis hospital, Medical Intensive Care Unit – Famirea group, AP-HP, 1 avenue Claude Vellefaux, 75010 Paris, France; Saint Louis Hospital, Biostatistics department, AP-HP, 1 avenue Claude Vellefaux, 75010 Paris, France; Biostatistics and Clinical Epidemiology, UMR 1153, INSERM, Paris Diderot Sorbonne University, Paris, France

**Keywords:** Intensive care, End of life, Condolence letter, Grief, Post-ICU syndrome

## Abstract

**Background:**

As intensive care mortality is high, end of life is a subject of major concern for intensivists. In this context, relatives are particularly vulnerable and prone to post-ICU syndrome, in the form of high levels of anxiety, depression, post-traumatic stress, and complicated grief. Grieving families suffer from a feeling of abandonment and evoke the need to get back in touch with the team to ask questions and remove doubts, but very few actually do. Aiding families during the grieving process is an important aspect of palliative care. A condolence letter represents an opportunity to recognize the pain of the family member and the strong tie that linked the family member to the ICU team, and to offer additional information if necessary. The goal of the study is to measure the impact of the condolence letter on the experience of bereaved families after a death in the ICU. Our hypothesis is that a post-death follow-up in the form of a condolence letter sent by the ICU physician who was in charge of the patient may help to reduce the risks of presenting symptoms of anxiety/depression, post-traumatic stress, and complicated grief.

**Methods/design:**

This is a randomized, controlled, multicenter study. Research will compare two groups of bereaved family members: one group that does not receive a condolence letter (control) and one group that receives a condolence letter 15 days after the death (intervention). Each of the 22 participating centers will include 12 relatives. Participating relatives will be followed up by phone with a call at 1 month and one at 6 months to complete questionnaires, permitting evaluation of post-ICU burden. The main outcome is anxiety and depression measured at 1 month. Other outcomes include evaluation of quality of dying and death, post-traumatic stress, and complicated grief.

**Discussion:**

This study will allow us to assess if sending a condolence letter can reduce the risks of presenting symptoms of anxiety and depression, complicated grief, and symptoms of post-traumatic stress disorder after the death of a loved one in the ICU.

**Trial registration:**

Clinical Trials registration number: Clinicaltrials.gov NCT02325297 (23 December 2014).

**Electronic supplementary material:**

The online version of this article (doi:10.1186/s13063-016-1212-9) contains supplementary material, which is available to authorized users.

## Background

### Context

As ICU mortality is high, end of life is a subject of major concern for intensivists. With a mortality rate of 20 % [1], end-of-life care has become a daily responsibility. Many deaths follow a decision to withhold or withdraw treatment, situations where physicians, nurses, and relatives must work together towards the most consensual decision. In the ICU, relatives are no longer simple visitors: they play active roles both at the patient’s bedside and with the team, thus creating a complex and unprecedented experience. A recent qualitative study showed that, after the loss, grieving families suffer from a feeling of abandonment by the ICU team [2]: “*During the end-of-life process you are cared for and then they sort of drop you*”. Indeed, the way families are cared for in the ICU is “intense”: daily contact with the nursing staff and physicians, precise information, phone calls, units that are open 24 hours a day. When the patient dies, families do not always have the opportunity to say goodbye to the team that cared for the patient, no longer receive attention from them, and therefore feel abandoned. Furthermore, for many family members, a few months after the death questions surface, incomprehension arises, doubts accumulate, and these questions remain without answer, provoking significant suffering and sometimes guilt. Many families evoke the need to get back in touch with the team to ask questions and remove doubts so as to ease the grieving process, but very few actually do.

A recent study from the Famiréa research group [3] shows that after the death of the patient in the ICU 52 % of family members present complicated grief symptoms at 6 months and 53 % at 12 months (using the ICG scale [4]) as well as a high risk of presenting post-traumatic stress symptoms (43.6 % at 6 months and 36.2 % at 12 months). The follow-up offered by the study (phone calls) was received as real support by the majority of families, a way of feeling not abandoned, the feeling that their pain counted, and that the ICU team continued to worry about them [2]. The Famiréa research group received numerous thank you letters. This brought us to consider the question of post-death follow-up and the interest of sending grieving family members a condolence letter.

### Condolence letters, a rare practice

Literature on follow-up with grieving families essentially concerns deaths in oncology units and in hospice care. These studies focus above all on the follow-up practices of oncology and hospice care physicians for grieving families: participation in rituals (funerals, for example), home visits, phone calls or condolence letters. Three studies [5–7] highlight heterogeneous and also very unpredictable practices: condolence letters written in 30–70 % of cases, few phone calls, little participation in grieving rituals. These three studies also highlight the barriers to condolence practices: lack of time or resources, the importance of maintaining professional distance, fear of professional burn-out, the difficulty of identifying which family member to contact, and, finally, the lack of recommendations for writing condolence letters. A literature review on the subject of interventions proposed to bereaved families demonstrates that many interventions are tested (from grief support groups to drug treatments to psychotherapy) without significant conclusions and that the majority of these studies suffer from significant methodological weaknesses [8]. Some studies ask the question of the identity of the person who could participate in the follow-up of grieving families: most often the doctor is cited and sometimes the nurse [9].

It is interesting to note that most studies focus on the practices of clinicians (oncologists and hospice care), but no study measures precisely the impact of these strategies on grieving individuals themselves. Two studies address this point, without it being the subject of the study, and demonstrate that a good interaction with the referring doctor of the dying patient, before and after his death, reduces complications in the grieving process [10] and, in the case of a phone call, diminishes the feelings of abandonment and solitude of family members [11]. Caring for family members after the death of a patient is one of the pillars of a good palliative approach. In its *Clinical Practice Guidelines for Quality Palliative Care*, the National Consensus Project for Quality Palliative Care [12] puts forward that among the nine points defining palliative care, one is dedicated to aiding families during their loved one’s illness and after death, during the grieving process. In the practical recommendation section on psychological care of patients and families, one section is dedicated to post-death follow-up and aiding grieving families during the first year of bereavement: after the patient’s death, family members may need help and follow-up allows them to be referred to professionals, if that is judged necessary, allows their pain to be recognized, and allows them to express themselves and to get back in touch with the care team if necessary. Therefore, contact with bereaved family members in the form of a condolence letter could be part of the care recommended by these guidelines.

### Condolence letter, whose responsibility?

The question of the physician’s responsibility is posed: When does the physician’s responsibility to his patient end? When the patient dies? Doesn’t care of the family constitute part of the physician’s responsibility to his patient? This reflection has fed some debates and publications, notably in the *New England Journal of Medicine* [13] and *Chest* [14]. Throughout these papers, a consensus appears: care doesn’t end at the death of the patient; it continues beyond with a last act of care, notably in the form of a condolence letter. “*A physician’s responsibility for the care of a patient does not end when the patient dies. There is one final responsibility — to help the bereaved family members. A letter of condolence can contribute to the healing of a bereaved family and help achieve closure in the relationship between the physician and the patient’s family*” [13]. The condolence letter not only allows family members to feel recognized and comforted but it also allows the caregiver who writes it to express his feelings and bring closure to a relationship. Finally, the practice of writing a condolence letter allows hospital units to orient their unit’s culture towards a more humanist approach to care and to paying attention to details in the relationship with the patient, the patient’s family, or even between the family and the patient: this is important for training young physicians and young nurses. Therefore, the condolence letter can be seen as a “*professional responsibility of the past that is important to revive*” [13].

### Recommendations

The literature allows us to better understand how to construct a condolence letter. Several points should be highlighted [13, 15]: the condolence letter should be short and handwritten; it should recognize the death and the pain of the loved one and bring him comfort; it should refer to the patient, or, if the physician didn’t know the patient well, to what the loved one did for the patient during his hospitalization (for example, his presence, participation in the care); there should be a reminder that the medical team remains available to answer questions and give the contact information of the referring physician; finally, the physician closes the letter with the presentation of his condolences, without being too formal: a “personal touch” is recommended.

The condolence letter, an obsolete practice these days in most medical specialties, appears to be the object of renewed interest. It is recommended both for bereaved family members and for the medical care team.

### Justification of the research project

To the best of our knowledge, there are no published studies that make reference to condolence letters (or any other practice used to follow up with bereaved family members) in the context of the ICU. Yet many studies have demonstrated the fragility of family members during the patient’s stay in the ICU and after discharge, notably if the patient dies. During the patient’s stay family members suffer from symptoms of anxiety and depression [16] as well as difficulty in understanding medical information [17].

The impact of the ICU stay on family members is real and long-lasting; a multicenter study done on 284 family members three months after the discharge or death of the patient demonstrates an important risk for family members. In fact, one-third of families show symptoms of post-traumatic stress disorder (PTSD). The predictive factors are [18] the quality of the information (the more the family members are dissatisfied, the greater the risk that they will present PTSD symptoms), and the death of the patient (the more the families feel they were implicated in the decision-making process for their loved one, the higher the risk of presenting symptoms of PTSD).

Strategies have been tested to reduce these long-term consequences, notably an end-of-life family conference that improves the experience of bereaved family members by reducing the risk of presenting PTSD 3 months after the patient’s death [19].

Finally, studies show on the one hand a higher risk of complications in the grieving process and a significant risk of PTSD and on the other hand that bereaved family members in this context need help, need to be recognized, need to be supported during their bereavement. A strategy must be developed to recognize both the pain of the family member and the strong tie that linked the family member to the ICU team.

### Objectives

The goal of the study is to measure the impact of the condolence letter on the experience of bereaved families after a death in the ICU: Does the condolence letter to bereaved family members reduce their risk of presenting symptoms of anxiety and depression and, later, symptoms of PTSD and complicated grief?

#### Hypothesis

Post-death follow-up in the form of a condolence letter sent by the intensive care physician who was in charge of the patient’s care may help to reduce the risks of presenting symptoms of anxiety/depression, post-traumatic stress, and complicated grief.

## Methods and design

### Population

Bereaved family members after the death of an adult patient in the ICU, fulfilling all inclusion criteria and none of the exclusion criteria.

Inclusion criteria

- Death of an adult (age ≥18) patient
- Death of a patient whose family has met the ICU team at least once before the death
- An ICU stay of at least two days

Non-inclusion criteria

- Death of a pregnant woman
- Family member who doesn’t speak French
- Refusal of the family member

As in previous Famiréa studies, only one family member is included: the “family representative,” that is, the designated health care proxy and, in his (her) absence, the family member most involved in the relationship with the ICU team, or by default the spouse (or partner), then the parents or children of the patient, then another member of the family.

### Methodology

This is a randomized, controlled, multicenter study. Research will compare two groups of bereaved family members: one group that does not receive a condolence letter and one group that receives a condolence letter 15 days after the death. Randomization will be stratified by center and balanced by permutation blocks (the size of which will not be communicated to the investigators). It will be done via a web server on a secure connection and will occur at inclusion (in the 24 hours following the death of the patient).

Family members will be included the day of the patient’s death (consent). The randomization (allowing the identification of family members of deceased patients who will receive a condolence letter) will occur after the consent by the family member to participate in the study—in the 24 hours following the death of the patient.

#### Primary endpoint

The HADS scale (Hospital Anxiety and Depression Scale) — 1 month after the death of the patient.

#### Secondary endpoints

**Fig. 1:**
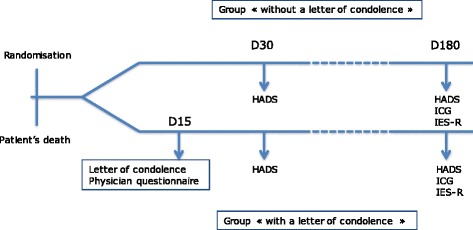
Study design

- HADS scale at 6 months after the death of the patient
- CAESAR scale measuring the experience of the end-of-life in the ICU — 1 month after the patient’s death
- IES-R (Impact of Event Scale, Revised) measuring the risk of presenting symptoms of post-traumatic stress — 6 months after the death of the patient
- ICG (Inventory of Complicated Grief) measuring the risk of presenting complicated grief symptoms — 6 months after the death of the patient
- Feasibility questionnaire for doctors who have written a condolence letter (Fig. 1)

#### Design

At the time of the patient’s death, the information letter (see Additional file 1) is given to the family member and consent to participate will be obtained. The same letter will be given to the two groups of family members; for ethical and psychological reasons, the condolence letter is not mentioned in the information letter. Once consent is obtained, relatives will be randomized to the intervention or the control group. For all the included patients, the physician will complete a “patient characteristics form.”

Control standard of care: no condolence letters to patients’ relatives

- All follow-up calls will be blinded: the caller will not know whether or not the family member received a condolence letter.
- Day 30: Call to the family member by the Famiréa group 1 month after the death to complete the HADS and the CAESAR scale.
- Day 180: Call to the family member by the Famiréa group 6 months after the death to complete the HADS, the ICG, and the IES-R.

Intervention: sending a condolence letter to patients’ relatives

- Day 1: in the 24 hours following the death, the physician in charge of the patient will write, with the help of the patient’s nurse, a condolence letter: it will be handwritten and written according to the recommendations of the group Famiréa. It will integrate the five following domains: 1) recognize the death and name the deceased; 2) mention the deceased; 3) recognize the family member; 4) offer help; 5) express sympathy (see Additional file 2). The letter will be photocopied and the original will be put in an envelope on which the family member’s address will be handwritten. The envelope will be placed in the physician’s office until it is sent via the post office. The photocopy of the letter will be placed in the case report form.

Following the writing of the letter, the physician will complete a questionnaire about the writing process (length of time, difficulties encountered, emotions).

- Day 15: The letter will be sent (in a hand-addressed envelope). An automatic alert (by email) generated by the randomization software will remind the physician to mail the condolence letter.
- Day 30: Call to the family member by the Famiréa group 1 month after the death to complete the HADS and the CAESAR scale.
- Day 180: Call to the family member by the group Famiréa 6 months after the death to complete the HADS, the ICG, and the IES-R.

In both the control and intervention groups, the reactions of the family member following the death of the patient will be screened. Did the family member call the physician? Did he (she) write to the team? Did he (she) come back to the unit? The screening will be done for 4 months following the death of the patient. Any letters written to the teams by the families during this period will be photocopied and sent to the Famiréa group.

### Data analysis

The analysis will be done according to the intention-to-treat principle (that is, each subject will be analyzed in the intervention group to which he (she) was assigned by the randomization, regardless of whether or not it was effectively done). We will report, in each randomization group, summary statistics according to the data (median and interquartile range, percentage with a 95 % confidence interval).

The proportion of family members with symptoms of anxiety/depression at days 30 and 180 will be compared between randomization arms by an exact Fisher test. A comparison of the HADS score distribution will be done using a non-parametric Wilcoxon rank sum test.

A regression model will be used later to search for associated factors should anxiety or depressive syndromes occur, or interactions between intervention effect and family member characteristics, notably the relationship (spouse, child) age, way of life.

Text data will also be analyzed with descriptive methods (factorial analysis of correspondences, word cloud), then predictive methods (text mining).

Statistical analysis will be done on software from SAS (SAS Inc., Cary, NC) and R (http://www.R-project.org/). All the tests will be two-sided with *p*-values of 0.05 or less indicating statistical significance.

### Justification of the sample size

On the basis of a previous Famiréa study [18], our hypothesis is that receiving a condolence letter could reduce the risk of presenting symptoms of anxiety/depression (HADS score) by 30 %, diminishing the prevalence at 1 month from 60 % in the group without a condolence letter to 42 % in the group with a condolence letter. In order to detect such a difference between the two groups, with a type I error of 0.05 and a power of 0.80, it is necessary to include 240 family members (120 in each group), that is, 12 inclusions per center in the 22 participating centers (see Additional file 3).

### Ethics

The study was approved by our local IRB (Comité de protection des personnes CPP Ile de France IV, Saint Louis, number 2014/14SC), the CNIL (Commission Nationale de l’Informatique et des Libertés), responsible for ensuring that information technology remains at the service of citizens, number MMS/VCS/AR 149697, and the CCTIRS (Comité Consultatif sur les Traitements de l’Information en matière de Recherche dans le domaine de la Santé), number 14.284.

## Discussion

This study will allow us to examine if sending a condolence letter can reduce the risks of presenting symptoms of anxiety and depression, complicated grief and symptoms of PTSD after a death in the ICU. If the result is positive, it would be possible to open this practice to all ICUs so as to help families in this sometimes complex and painful process. This practice has no financial costs. Reducing rates of anxiety, depression, post-traumatic stress, and complicated grief after a death in the ICU is important as a public health issue: people suffering from these symptoms require medical care, follow-up, and treatment. Reducing the rate of post-ICU burden would allow a reduction of medical consultations and treatments. The results of the study would allow other hospital units to reflect on putting a similar strategy in place.

### Trial status

The trial is currently recruiting patients. Inclusion started on 1 December 2014.

## Additional files

Additional file 1:
**Family Information Letter.** (DOCX 16 kb) Additional file 2:
**Recommendations for writing a condolence letter and examples.** (DOCX 22 kb) Additional file 3:
**Lists of centers participating in the Famiréa 22 study.** (DOCX 18 kb)

## Abbreviations

HADS: Hospital Anxiety and Depression Scale
ICG: Inventory of Complicated Grief
ICU: intensive care unit
IES-R: Impact of Event Scale-Revised
