# Supplementary material for: Impact of the condolence letter on the experience of bereaved families after a death in intensive care: study protocol for a randomized controlled trial
Source: Trials. 2016 Feb 20;17:102. doi: 10.1186/s13063-016-1212-9 (PMC4761130; doi:10.1186/s13063-016-1212-9)
Supplement: Additional file 1: — Family Information Letter. (DOCX 16 kb) [file 13063_2016_1212_MOESM1_ESM.docx]

**Additional file 1**

Family Information Letter

**Information Letter**

Famiréa Study XXII

Madam, Sir,

Your family member died in Intensive Care and we present our most sincere condolences. We know that the death of a loved one is one of the most difficult life experiences and our whole team joins you in your grief.

In the context of a national study in several Intensive Care units, we seek to understand how the grieving process unfolds after a death in our unit. Your participation in this research program would be precious to help us improve the quality of our care for patients admitted into Intensive Care.

Naturally you have the right to refuse to participate in this study. If that is the case, inform the physician of your refusal. If you accept to participate, you can, at any moment, exercise your right to withdraw from the research by contacting the research group Famiréa at 01 42 49 94 24.

Participation in this study only requires a few phone interviews with a member of the research group. If you accept, you will receive a first phone call in one month during which you will complete a questionnaire to tell us how you feel. We will contact you again in six months by mail and in eight months for another phone conversation. The file of your personal information will be destroyed after your participation in this research. In conformity with the law on personal digital data, you have the right of access and of correction (contact the group Famiréa at the number above). You also have the right to oppose the transmission of data covered by professional confidentiality laws which might be used in the context of this study.

We thank you for your understanding and your participation in this research project.

The Famiréa^^[[1]](#footnote-1)^*^ Research Group

1. * Famiréa is a pluridisciplinary research group based at the Hôpital Saint Louis, in Paris, whose studies cover the experience of families and patients with the goal of improving care practices, communication and the experience of all the participants in Intensive Care. [↑](#footnote-ref-1)
