# Supplementary material for: Impact of the condolence letter on the experience of bereaved families after a death in intensive care: study protocol for a randomized controlled trial
Source: Trials. 2016 Feb 20;17:102. doi: 10.1186/s13063-016-1212-9 (PMC4761130; doi:10.1186/s13063-016-1212-9)
Supplement: Additional file 2: — Recommendations for writing a condolence letter and examples. (DOCX 22 kb) [file 13063_2016_1212_MOESM2_ESM.docx]

**Additional file 2**

Recommendations for writing a condolence letter and examples

**Why write a condolence letter?**

- To help family members in the bereavement process: the letter helps relatives feel recognized in their pain and not abandoned by the hospital team
- To help family members manage potential feelings of anger or lack of understanding following an unexpected death
- To help the physician take stock of the patient’s death
- To bring closure to the relationship between caregivers and the families of the deceased patient

**Recommendations for writing a condolence letter**

***THE CONDOLENCE LETTER MUST BE HANDWRITTEN***

- Avoid superficial expressions like “I know what you’re feeling”
- Don’t write too formal a letter!
- Please be sure to integrate the following five domains:

***5 DOMAINS TO INCLUDE IN THE LETTER:***

1. **Recognize the death – Name the deceased**

- The importance of naming the deceased
- Reduces the feeling of solitude of the family member

1. **Talk about the deceased**

- If possible: his personality, his age, his interests (sports, religion…)
- If possible, mention a specific memory of the deceased
- If possible, mention his relationship with the family member

1. **Recognize the family member**

- His personality, his strengths (to reinforce his feeling that he can manage)
- What the family member did for or with the patient in ICU (frequent visits, participating in care, etc.)
- Or even his relationship with the team

1. **Offer help: the possibility of contacting you**

- Be specific (the phone number of the unit)

1. **Express your sympathy (conclusion)**

- Symbolising a shared emotion

**Examples**

1. **Recognize the death and name the deceased**

I send you my sincere condolences on the death of **your brother, Alfred Smith**. Natalie, who was your brother’s nurse, joins me in expressing our sympathy.

1. **Mention the deceased**
2. ***Patient who was conscious and able to communicate:***

We had the opportunity to get to know your brother during his stay on our unit. He was very **brave. His smile and his words** touched us often. His caregivers were always happy to go into his room.

*Or*

We had the opportunity to get to know your brother during his stay on our unit. He was very **brave**. We understood his **need to be cared for and reassured** and we hope we were able to comfort him in the difficult moments.

1. ***Patient who was conscious but had difficulty communicating:***

We had the opportunity to get to know your brother during his stay in our unit. He seemed very **brave**. He tried to communicate with us in different ways, for example using the whiteboard we gave him, even though we know it was sometimes difficult for him.

1. ***Patient who was never conscious in Intensive Care***

We didn’t have the opportunity to really get to know your brother and we regret that. However, thanks to his family members, we could see that he was **a kind and brave man** and we did our best to care for him and aid him with kindness and respect.

1. **Recognize the family member**

**You were very present** during his hospitalisation, ready to assist and be present for your brother. In my experience as a physician**, I believe that the presence and support of a family member brings peace and serenity to those who are at the end of life.**

1. **Offer help**

**I remain at your service** if you wish to ask any questions or simply discuss your brother’s stay in Intensive care. Please don’t hesitate to call us at [telephone number].

1. **Express your sympathy (conclusion)**

**We send you our warmest thoughts,**

Dr Doe.
