# Supplementary material for: Impact of the condolence letter on the experience of bereaved families after a death in intensive care: study protocol for a randomized controlled trial
Source: Trials. 2016 Feb 20;17:102. doi: 10.1186/s13063-016-1212-9 (PMC4761130; doi:10.1186/s13063-016-1212-9)
Supplement: Additional file 3: — Lists of centers participating in the Famiréa 22 study. (DOCX 18 kb) [file 13063_2016_1212_MOESM3_ESM.docx]

**Additional file 3**

Lists of centers participating in the Famiréa 22 study

|  | **Name of the investigator** | **City** | **Hospital** | **Type of Intensive Care** |
| --- | --- | --- | --- | --- |
| 1 | **VENOT** Marion | Paris | Saint Louis | Medical Intensive Care |
| 2 | **CHAMPIGNEULLE** Benoît | Paris | Cochin | Medical Intensive Care |
| 3 | **GARROUSTE** Maité | Paris | Saint Joseph | General Intensive Care |
| 4 | **TROCHE** Gilles | Le Chesnay-Versailles | André Mignot | General Intensive Care |
| 5 | **GUISSET** Olivier | Bordeaux | Saint André | Medical Intensive Care |
| 6 | **RENAULT** Anne | Brest | Cavale Blanche | Medical Intensive Care |
| 7 | **ARGAUD** Laurent | Lyon | Edouard Herriot | Medical Intensive Care |
| 8 | **ADDA Mélanie** | Marseille | Hôpital Nord | Medical Intensive Care |
| 9 | **RIGAUD** Jean-  Philippe | Dieppe | CH de Dieppe | General Intensive Care |
| 10 | **VINATIER** Isabelle | La Roche-sur-Yon | Les Oudairies | General Intensive Care |
| 11 | **CARR** Julie | Montpellier | Saint Eloi | General Intensive Care |
| 12 | **THIRION** Marina | Argenteuil | CH Victor Dupouy | General Intensive Care |
| 13 | **LESIEUR** Olivier | La Rochelle | CH de la Rochelle | General Intensive Care |
| 14 | **ROBERT** René | Poitiers | CHU de Poitiers | Medical Intensive Care |
| 15 | **CINOTTI** Raphaël | Nantes | CHU de Nantes | Surgical Intensive Care |
| 16 | **SOUWEINE** Bertrand | Clermont Ferrand | CHU Gabriel Montpied | General Intensive Care |
| 17 | **BORNSTAIN** Caroline | Montfermeil | CHI Le Raincy | General Intensive Care |
| 18 | **GILBERT Marion** | Corbeil | CH Sud-Francilien | General Intensive Care |
| 19 | **GADAY** Véronique | Pontoise | CH René Dubos | General Intensive Care |
| 20 | **DEMOULE** Alexandre | Paris | La Pitié-Salpêtrière | Medical Intensive Care |
| 21 | **SLAMA** Michel | Amiens | CHU Amiens-Picardie Hôp Sud | Nephrology Intensive Care |
| 22 | **MASSOT** Julien | Paris | HEGP | Anesthesia – Surgical Intensive Care |
